# An Ensemble Learning Method Based on an Evidential Reasoning Rule considering Combination Weighting

**DOI:** 10.1155/2022/1156748

**Published:** 2022-03-07

**Authors:** Cong Xu, YunYi Zhang, Wei Zhang, HongQuan Zu, YiZhe Zhang, Wei He

**Affiliations:** ^1^Harbin Normal University, Harbin 150025, China; ^2^Harbin Institute of Technology, Harbin 150025, China; ^3^Rocket Force University of Engineering, Xi'an 710025, China

## Abstract

As an extension of Dempster–Shafer (D-S) theory, the evidential reasoning (ER) rule can be used as a combination strategy in ensemble learning to deeply mine classifier information through decision-making reasoning. The weight of evidence is an important parameter in the ER rule, which has a significant effect on the result of ensemble learning. However, current research results on the weight of evidence are not ideal, leveraging expert knowledge to assign weights leads to the excessive subjectivity, and using sample statistical methods to assign weights relies too heavily on the samples, so the determined weights sometimes differ greatly from the actual importance of the attributes. Therefore, to solve the problem of excessive subjectivity and objectivity of the weights of evidence, and further improve the accuracy of ensemble learning based on the ER rule, we propose a novel combination weighting method to determine the weight of evidence. The combined weights are calculated by leveraging our proposed method to combine subjective and objective weights of evidence. The regularization of these weights is studied. Then, the evidential reasoning rule is used to integrate different classifiers. Five case studies of image classification datasets have been conducted to demonstrate the effectiveness of the combination weighting method.

## 1. Introduction

Ensemble learning is a branch of machine learning, and using decision-making reasoning as a combination strategy helps ensemble learning to integrate better. The decision-making reasoning is the process of making a choice among many options and summarizing evidence to draw a conclusion, so it can help ensemble learning to integrate. The ER rule is a combination strategy [1], which can integrate classifiers through decision-making reasoning to obtain better results, so it is used as a decision-making strategy for ensemble learning in this paper.

In 2013, the ER rule, which considers the weight and reliability of evidence, was established by Yang and Xu [1]. The ER rule, which is an extension of the D-S theory [2, 3], clearly distinguishes the importance and reliability of evidence and constitutes a general joint probabilistic reasoning process. The counterintuitive problem encountered in Dempster's rule is solved by assigning weight and reliability to evidence. Due to the superiority of the ER rule, it has been applied in many different applications. A fault detection method based on the data reliability and interval ER rule was proposed, which improved the accuracy of fault detection [4]. A multi-index framework was proposed based on the combination of objective quality indicators, subjective expert judgements, and patient feedback based on the ER rule and improved model robustness under uncertain conditions [5]. The above research provides a theoretical basis and technical support for the research and application of the ER rule. The ER rule has become an important element in the field of nonclassical reasoning and can deeply mine classifier information through decision-making reasoning and improve the integration model.

The weight and reliability of evidence can be as key parameters in the ER rule have been extensively studied in existing research. There are generally three ways to set the weight of evidence in the ER rule. First, the weight of evidence in the traditional ER rule is given by expert knowledge. For example, the order relation analysis method (G1) was used to quantitatively evaluate the comprehensive intelligence of autonomous vehicles [6]. The weights used in the literature [7, 8] were assigned by experts who actually assessed indicators from industrial processes. But this kind of human subjective weighting method is overpowering and prone to deviation in results. Second, some researchers have recently improved the setting of the weight of evidence. For example, Wu et al. [9] proposed popular calibration weighting methods such as generalized regression and generalized exponential tilting and verified their effectiveness. Three methods of the maximum deviation method, coefficient of variation method, and entropy weight method were combined to establish an index optimal adaptive weighting model [10]. A novel weighted majority voting ensemble (WMVE) approach was proposed to assign different weights to classifiers [11]. A comprehensive evaluation method of power quality based on the ratio of the weighted rank sum ratio method was proposed to correctly sort and classify the power quality of each bus [12]. Zhou et al. [13] carried out physical and chemical analyses and used the coefficient of variation, mean and standard deviation in a model to predict the proximate and ultimate analysis and heating value from physical compositions. These methods assigned weights in an objective way. But sometimes, heavily relying on the sample causes the weight setting to be inconsistent with the actual situation. Third, mathematical optimization was adopted to produce a dual-objective optimization model that was trained to obtain the weight of diagnostic evidence [14]. However, the mathematical optimization method needs a large number of samples, and it is difficult to obtain enough training samples in practice. Most of the proposed weighting methods are only applicable to a specific engineering background and are not universal. There is still a lack of a general method to determine the weight of evidence. Therefore, to set a more general and reasonable evidence weight, this paper proposes to use the combination weighting method to set the weight of evidence in the ER rule.

Combination weighting is a method that combines subjective weighting and objective weighting. This method can minimize the loss of information and can reflect both subjective and objective information, thereby making the determined weights more reasonable. At present, the combination weighting method has been widely used to weight the risk factors for fault modes to determine the risk priority of fault mode recognition [15]. This method was also used to determine the assessment factors of project managers, and a new method of manager performance evaluation was proposed [16]. The game theory method was proposed to combine the subjective weighting method and objective weighting method. The evaluation system of the educational informatization index was established [17], and it was verified in the Suzhou area. An et al. used the IGAHP-CRITIC combination weighting method to effectively integrate subjective and objective weight information, which effectively reduced the uncertainty and incompleteness of an equipment quality state assessment [18]. Since the setting of weights directly affects the accuracy of integrated learning using the ER rule, the complexity of objective things causes objective weighting to rely heavily on samples, and subjective weighting may lead to inaccurate weight settings due to the ambiguity of personal thought processes. Therefore, research in the literature [19] verified the rationality of the combination weighting of the maximum difference by comparing the combination weighting method of addition, multiplication and the combination weighting method of maximum difference and provided a new idea for the combination of subjective and objective weights. The weighting method of maximizing the difference is used to evaluate the operation status of the electricity market in the literature [20]. This method achieved excellent results, reflecting its practicality and innovation. The effectiveness of the combination weighting method was proven by these examples.

The application of the ER rule to ensemble learning is a new research field in the context of artificial intelligence. In ensemble learning, the generation of classifiers and the choice of combination strategies have a great impact on the quality of the results [21]. At present, a variety of methods have been applied as a combination strategy in the field of integrated learning, such as the voting method [22], average method [23], learning method [24], and D-S theory [25–27]. However, these methods have difficulty effectively mining the relationship between classifiers, and the ability to integrate classifiers is limited. Based on its advantages in improving uncertainty and reasoning, the ER rule can allow integrated models to obtain better results than strategies such as voting. Additionally, the ER rule does not change the traditional probabilistic reasoning model, so it is more effective for classifiers to integrate this kind of uncertainty information. As a powerful engine and information fusion mechanism [1], the ER rule can effectively improve the effect of ensemble learning.

In summary, a new ensemble learning model is constructed based on the ER rule. The contributions of this paper are described as follows:

1. The combination weighting method is used to determine the weight of evidence in the ER rule. It can further find the effective information in the process of combining evidence, take subjective factors and objective factors of evidence into consideration, and set more general and reasonable weight of evidence for the ER rule.
2. The ER rule is an information fusion method that does not contain labels. When setting weights, whether it is necessary to regularize the combination of weights has always been a matter of debate among existing researches. Accuracy is used as an indicator to study the regularization of weights in the ER rule and our own suggestions are given in this paper.
3. An ensemble learning method is proposed based on the ER rule considering combination weighting, and it can effectively improve the effect of ensemble learning.

The rest of this paper is organized as follows: using combination weighting method to determine weights for the ensemble learning model based on the ER rule is proposed in Section 2. The combination weighting method is constructed in Section 3. The ensemble learning model based on the ER rule is constructed in Section 4. Cases are used to verify the effectiveness of the combination weighting method is presented in Section 5. The conclusion of this paper is provided in Section 6.

## 2. Problem Definition

In the application of ensemble learning based on the ER rule, to demonstrate that the ER rule has a better improvement effect on the ensemble learning model, it is necessary to adopt appropriate weighting methods to set the weight of evidence. The main problems to be solved in this paper are as follows:

**Problem 1 .**

construction of the combination weighting model and regularization of weight). At present, when assigning the weight of each classifier in ensemble learning, each classifier is used as a piece of evidence. Classifiers are usually generated by black-box models and are not interpretable, so it is difficult to provide accurate weight values through expert knowledge. In addition, the number of classifiers used in ensemble learning is small, and it is difficult to determine the weights in a mathematically optimized way. In the commonly used weighting method, the subjective weighting method is strongly influenced by human factors, and the objective weighting method depends a lot on samples. Therefore, subjective weighting and objective weighting are combined in this paper. We established a combination weighting model for weights:

(1) $$\begin{array}{c} \omega_{k}=F\left(\overset{⟶}{\omega_{k}},\overset{⟵}{\omega_{k}},\gamma \right), \end{array}$$

where *ω*_*k*_ is the combined weight of the classifier *k*, *F*(•) is a combination method of subjective weights and objective weights, $\overset{⟶}{\omega_{k}}$ is the subjective weight of the classifier *k* calculated by the subjective weighting method, $\overset{⟶}{\omega_{k}}$ is the objective weight of the classifier *k* calculated by the objective weighting method, and *γ* is the parameter combination of the weight combination process. It is uncertain whether different combination weighting methods can make use of the advantages of subjective weights and objective weights, which needs to be verified in studies. The weight determined by the combination weighting method may exceed the range of *ω*_*k*_. For weight combinations that exceed the range of *ω*_*k*_, it is necessary to regularize this weight, but for weight combinations that do not exceed the range of *ω*_*k*_, whether regularization is required has always been a controversial issue when using the ER rule. Assuming there are *n* classifiers in total, the regularization process of weights can be described as

(2) $$\begin{array}{c} \bar{\omega_{k}}=\frac{\omega_{k}}{\sum_{i=1}^{n}\omega_{i}},\;k=1,2,\ldots ,n, \end{array}$$

where $\bar{\omega_{k}}$ is the regularized result of weight *ω*_*k*_ and *ω*_*k*_ is the combined weight of the classifier *k*.

**Problem 2 .**

(construction of the ensemble learning model based on the ER rule). In the current ensemble learning research, how to use a suitable combination strategy to combine weak learners into strong learners is the main problem. As an ensemble strategy, the ER rule can consider the relationship between classifiers, overcome the shortcomings of algorithms that only consider the numerical relationship of the classifier data, and significantly improve the effect of ensemble learning. Suppose a total of *K* classifiers are integrated, and the process of the ensemble learning model based on the ER rule is as follows:

(3) $$\begin{array}{c} c\left(k\right)=f_{k}\left(d,\tau \right), \end{array}$$

where *c*(*k*) is the classification result of the classifier *k*, *k*=1,2,…, *K*, *f*_*k*_(*·*) is the classification process of the classifier *k*, *d* is the dataset, and *τ* is the parameter set in the classification process. After the classification result is obtained, the ensemble result *u* of the model can be described as

(4) $$\begin{array}{c} u=g\left(C\left(K\right),\psi \right), \end{array}$$

where *g*(*·*) is the integration process of the model, *C*(*K*) is the set of classification results of *K* classifiers, and *ψ* is the parameter set in the ensemble process.

**Figure 1:**
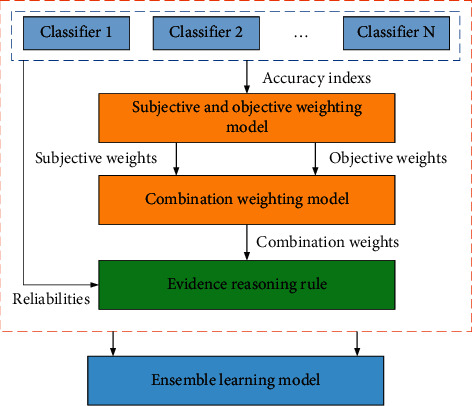
Ensemble learning model based on the ER rule considering combination weighting.

To solve the above problems, an ensemble learning model based on the ER rule considering combination weighting is proposed in this paper, as shown in Figure 1. It consists of two parts: combination weighting and the ER rule, which are introduced in detail in Section 3 and Section 4, respectively.

## 3. Construction of Weighting Methods

The weight of evidence is an important part of the ER rule, and the weighting method is the focus of this paper. Some representative weighting methods [28] are selected for definition in this section. The weights identified in this section will be used in Section 4.2.

### 3.1. Subjective Weighting

The subjective weighting method is a method of weighting based on the subjective information of the decision-maker. This method can reflect the degree of importance the decision-maker attached to different attributes and can flexibly grasp the importance of each decision-making attribute. However, its flexibility and variability can carry too much subjective arbitrariness. The subjective weighting method chosen in this paper is the analytic hierarchy process (AHP).

#### 3.1.1. Analytic Hierarchy Process

The analytic hierarchy process is based on the experience of the decision-maker, combining quantitative analysis with qualitative analysis, judging the relative importance of each measurement target, and providing the weight of the decision plan.

The first step is to build a hierarchical structure model. The relevant factors are decomposed into several levels from the top to bottom according to different attributes, and the goals, criteria, and objects of decision-making are divided into the highest level, the middle level, and the lowest level, respectively.

The second step is that a judgement matrix is constructed. Based on the accuracy of each classifier, the classifiers are compared in pairs, a scale is given, and a matrix is formed.

The third step is hierarchical single sorting and consistency checking. Hierarchical single sorting calculates the importance weight of the index related to the level for the index of the upper level according to the judgement matrix. Then, a check is provided on whether the judgement matrix is a consistent matrix to determine the weight of the classifier.

The fourth step is that the total taxi of hierarchy occurs, and consistency is checked. After calculating the comprehensive weights of all classifiers, they are sorted to obtain the importance results.

### 3.2. Objective Weighting

The objective weighting method refers to the method of using the objective information of each attribute to determine the attribute weight. The decisions or evaluation results have a strong theoretical basis. However, the objective weighting method cannot address the subjective wishes of the decision-maker, which sometimes it goes against the actual importance of the attribute. The objective weighting methods used in this paper are the entropy weight method (EW), coefficient of variation method (COV), and CRITIC method.

#### 3.2.1. Entropy Weight Method

The entropy weight method is based on the amount of information contained in the data in the evaluation index system and determines the index weights by calculating the information entropy. The calculation steps are as follows.

Suppose there are *I* evaluation samples, *K* classifiers, and *T* categories. Then there will be *K* × *T* evaluation indicators *X*_1_, *X*_2_,…, *X*_*KT*_, *X*_*j*_={*x*_1*j*_, *x*_2*j*_,…, *x*_*Ij*_}, and *x*_*ij*_ is the *j* index value of the *i* samples (*i*=1,2,…, *I*;  *j*=1,2,…, *KT*); the indicators used in this paper are all positive and the value *y*_*ij*_ of the evaluation index is calculated through the standardization of Min − Max:

(5) $$\begin{array}{c} y_{ij}=\left\{\begin{array}{cc} 0 & \max\left(X_{j}\right)=\min\left(X_{j}\right)\\ \frac{x_{ij}-\min\left(X_{j}\right)}{\max\left(X_{j}\right)-\min\left(X_{j}\right)} & \max\left(X_{j}\right)\min\left(X_{j}\right) \end{array}\right). \end{array}$$

Then, according to the evaluation index value *y*_*ij*_, the standardization method is used to construct the evaluation matrix *Y*_*ij*_, and the process is shown in the following equation:

(6) $$\begin{array}{c} Y_{ij}=\left\{\begin{array}{cc} 0 & \max\left(X_{j}\right)=\min\left(X_{j}\right)\\ \frac{y_{ij}}{\sum_{i}^{I}y_{ij}} & \max\left(X_{j}\right)\min\left(X_{j}\right) \end{array}\right). \end{array}$$

After obtaining the standardized positive indicators, then their information entropy is computed. The calculation of the information entropy *E*_*j*_ of the index *j* in the matrix is shown in the following equation:

(7) $$\begin{array}{c} E_{j}=\left\{\begin{array}{cc} 0 & \max\left(X_{j}\right)=\min\left(X_{j}\right)\\ -\ln\;{\left(I\right)}^{-1}\sum_{i}^{I}Y_{ij}\ln\;Y_{ij} & \max\left(X_{j}\right)\min\left(X_{j}\right) \end{array}\right). \end{array}$$

The normalization method is used to determine the weight of the abovementioned positive indicators. Since there are *K* classifiers and *T* categories, there are a total of *K* × *T* positive indicators. The information entropy of each group of data calculated by Equation (7) is: *E*_1_, *E*_2_,…, *E*_*KT*_, and the indicator weight is calculated by using Equation (8) to calculate the information entropy.

(8) $$\begin{array}{c} \omega_{kt}=\omega_{j}=\frac{1-E_{j}}{I-\sum_{j=1}^{KT}E_{j}}, \end{array}$$

where *ω*_*kt*_ is the weight of the indicator *kt*. Therefore, the weight determined by the entropy weight method of the *k* classifier can be expressed as *ω*_*k*_, and each classifier recognizes the data of *T* categories to obtain *T* weight values. The weight of the index *kt* is calculated by using Equation (8). Therefore, the weight of the classifier *k* is calculated by

(9) $$\begin{array}{c} \omega_{k}=\sum_{t=1}^{T}\omega_{kt}. \end{array}$$

#### 3.2.2. Coefficient of Variation Method

The coefficient of variation method is based on the degree of variation between the current value of each index in the evaluation index system and the target value, and the index weight is determined by the method of calculating the degree of change of each index in the system. The calculation steps are as follows.

The steps in the entropy weight method in Section 3.2.1. Assuming there are *I* evaluation samples, *K* classifiers, and *T* categories, there are *K* × *T* evaluation indicators. The evaluation index *y*_*ij*_ is calculated by Equation (5).

After the evaluation index *y*_*ij*_ is calculated, the mean value and standard deviation of the evaluation index are also calculated, and the coefficient of variation is calculated through the mean value and standard deviation of the index. The calculation process of the coefficient of variation *v*_*j*_ of the index *j* in the matrix is as follows:

(10) $$\begin{array}{c} v_{j}=\frac{\sqrt{\left(\sum_{i=1}^{I}\left(y_{ij}-\left(1/I\right)\sum_{i=1}^{I}y_{ij}{}^{2}\right)/I-1\right)}}{\left(1/I\right)\sum_{i=1}^{I}y_{ij}},\;j=1,2,\ldots ,KT, \end{array}$$

where $\sqrt{\sum_{i=1}^{I}\left(y_{ij}-\left(1/I\right)\sum_{i=1}^{I}y_{ij}{}^{2}\right)/\left(I-1\right)}$ is the standard deviation of the evaluation index *j* and 1/*I*∑_*i*=1_^*I*^*y*_*ij*_ is the average value of the evaluation index *j*. Then the coefficient of variation is normalized, and the weight of each evaluation index is calculated as follows:

(11) $$\begin{array}{c} \omega_{kt}=\omega_{j}=\frac{v_{j}}{\sum_{j=1}^{KT}v_{j}}, \end{array}$$

where *ω*_*kt*_ is the weight of the indicator *kt*. The weight determined by the coefficient of variation method for classifier *k* can be expressed as *ω*_*k*_, which can be calculated by Equation (9).

#### 3.2.3. CRITIC Method

The CRITIC method is based on the contrast strength and conflict of each index in the evaluation index system, and it determines the index weight through the objective attributes of the data itself. The calculation steps are as follows:

The steps in the entropy weight method in Section 3.2.1 are also applied here. The evaluation index *y*_*ij*_ is calculated by Equation (5). Then the amount of information *C*_*j*_ of the index *j* in the matrix is calculated:

(12) $$\begin{array}{c} C_{j}=\sqrt{\frac{\sum_{i=1}^{I}\left(y_{ij}-\left(1/I\right)\sum_{i=1}^{I}y_{ij}{}^{2}\right)}{I-1}}\sum_{i=1}^{KT}\left(1-r_{ij}\right), \end{array}$$

where $\sqrt{\sum_{i=1}^{I}\left(y_{ij}-\left(1/I\right)\sum_{i=1}^{I}y_{ij}{}^{2}\right)/\left(I-1\right)}$ is the standard deviation of the index *j* and *r*_*ij*_ is the correlation coefficient between index *i* and index *j*.The correlation coefficient is used to express the correlation between indicators. If the correlation between an indicator and other indicators is stronger, the conflict will be smaller, and the weight assigned to the indicator should be reduced. The weight of evaluation indicator is as follows:

(13) $$\begin{array}{c} \omega_{kt}=\omega_{j}=\frac{C_{j}}{\sum_{j=1}^{KT}C_{j}}, \end{array}$$

where *ω*_*kt*_ is the weight of the indicator *kt*.The weight *ω*_*k*_ of classifier *k* can be calculated by Equation (9).

In view of the shortcomings of the above methods, the combination weighting method can effectively solve the problem of too strong subjectivity or objectivity of a single weighting method. At the same time, no one had used the combination weighting method to determine the weight of evidence of the ER rule. Therefore, the combination weighting method is defined in Section 3.3.

### 3.3. Combination Weighting

**Figure 2:**
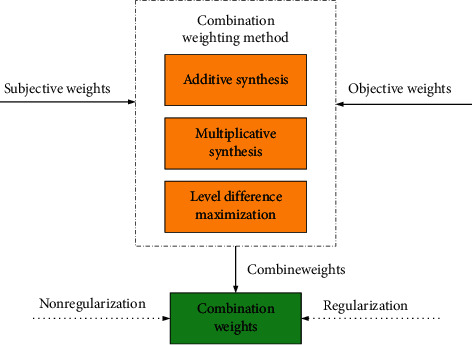
The process of weights combination.

Combination weighting can effectively address the subjective and objective information of attributes, so it is more reasonable for setting the weight of evidence. This paper uses three combination weighting methods, including additive synthesis, multiplicative synthesis and level difference maximization. After the combined weights are determined, they need to be regularized. The weighting process of the three combination weighting methods is shown in Figure 2.

#### 3.3.1. Research on Additive Synthesis

The combined weights based on the additive synthesis method mainly depend on the distribution of the subjective weights and the weight coefficient of the objective weights. At present, the commonly used methods include the minimum weighting method of subjective and objective weight deviation. This paper only studies the basic weight accumulation. Assuming there are *n* classifiers, the combination of subjective weight and objective weight of the classifier *k* can be described as

(14) $$\begin{array}{c} \omega_{k}:\;\text{if }0\leq \omega_{k}\leq 1,\\ \text{then }\omega_{k}\text{ is preserved,}\\ \text{else}\\ \text{then }\omega_{k}⟶\bar{\omega_{k}}=\frac{\omega_{k}}{\sum_{i=1}^{n}\omega_{i}},\;k=1,2,\ldots ,n\text{ as the new weight,}\\ \omega_{k}=\overset{⟶}{\omega_{k}}+\overset{⟵}{\omega_{k}}. \end{array}$$

The subjective weight ${\overset{⟶}{\omega }}_{k}$ and the objective weight ${\overset{⟵}{\omega }}_{k}$ of classifier *k* are added to obtain the combined weight *ω*_*k*_, and then it is judged whether *ω*_*k*_ satisfies 0 ≤ *ω*_*k*_ ≤ 1. If the combined weight *ω*_*k*_ satisfies the condition, it will be retained, otherwise it will be regularized.

#### 3.3.2. Research on Multiplicative Synthesis Method

The multiplicative synthesis method has a multiplier effect, which leads to a higher combination weight of a classifier with a higher subjective and objective weight, and a lower combination weight with a smaller weight. Assuming there are *n* classifiers, the combination of subjective weight and objective weight of the classifier *k* can be described as

(15) $$\begin{array}{c} \omega_{k}:\;\text{if }0\leq \omega_{k}\leq 1,\\ \text{then }\omega_{k}\text{ is preserved,}\\ \text{else}\\ \text{then }\omega_{k}⟶\bar{\omega_{k}}=\frac{\omega_{k}}{\sum_{i=1}^{n}\omega_{i}},\;k=1,2,\ldots ,n\text{ as the new weight,}\\ \omega_{k}=\overset{⟶}{\omega_{k}}*\overset{⟵}{\omega_{k}}. \end{array}$$

The subjective weight $\overset{⟶}{\omega_{k}}$ and the objective weight $\overset{⟵}{\omega_{k}}$ of classifier *k* multiplied to obtain the combined weight *ω*_*k*_, and then it is judged on whether *ω*_*k*_ satisfies 0 ≤ *ω*_*k*_ ≤ 1. If the combined weight *ω*_*k*_ satisfies the condition, it will be retained, otherwise it will be regularized.

#### 3.3.3. Research on the Level Difference Maximization

The combination weighting method with level difference maximization is a combination weighting method with a single index as the combined unit [29]. First, the multiple subjective weights and objective weights of the classifier are computed according to different weighting methods. Then, the reasonable value space of the combined weights is determined according to the multiple weights; Finally, the maximum discrimination of the evaluation results is taken as the objective function, and the index's reasonable value interval of the weight is used to establish an optimization model for the constraint conditions and solve the combined weight of the evaluation index. The detailed description of the method is as follows:

Suppose the scheme set is *X*=(*x*_1_, *x*_2_,…, *x*_3_) and the attribute set is *G*=(*g*_1_, *g*_2_,…, *g*_*m*_). Then, *y*_*ij*_=*g*_*i*_(*x*_*j*_) is the attribute value of scheme *x*_*j*_ under attribute *g*_*i*_, *r*_*ij*_ is the normalized result of the decision matrix *Y*=(*y*_*ij*_)_*m*×*n*_, the weight vector of the attribute is *ω*=(*ω*_1_, *ω*_2_,…,*ω*_*n*_)^*τ*^, and *ω*_*i*_ ≥ 0, ∑_*i*=1_^*n*^*ω*_*i*_=1. Thus, the comprehensive attribute value of scheme *x*_*j*_ can be calculated:

(16) $$\begin{array}{c} r_{j}=\sum_{i=1}^{m}\omega_{i}r_{ij},\;j\in N. \end{array}$$

Attribute *X* represents the total variance of all decision-making plans and other decision-making plans, and the weight vector *X* should be selected to maximize the total variance of all the attributes for all decision-making plans, so that the deviation function can be constructed:

(17) $$\begin{array}{c} \sigma \left(\omega \right)=\sum_{i=1}^{m}\sigma_{i}\left(\omega \right)=\sum_{i=1}^{m}\sum_{j=1}^{n}{\left(\omega_{i}r_{ij}-\omega_{i}\bar{r_{i}}\right)}^{2}=\sum_{i=1}^{m}\sum_{j=1}^{n}{\left(r_{ij}-\bar{r_{i}}\right)}^{2}\omega_{i}^{2}. \end{array}$$

Thus, by solving the following single-objective optimization problem model, the weight vector of the attribute can be calculated:

(18) $$\begin{array}{c} s.t.\sum_{i=1}^{m}\omega_{i}=1,\;\omega_{i}\geq 0,\;i\in M,\\ \max\sigma \left(\omega \right)=\sum_{i=1}^{m}\sum_{j=1}^{n}{\left(r_{ij}-\bar{r_{i}}\right)}^{2}\omega_{i}^{2}. \end{array}$$

In this paper, the level difference maximization is described as

(19) $$\begin{array}{c} \omega_{k}:\;\text{if }0\leq \omega_{k}\leq 1,\\ \text{then }\omega_{k}\text{ is preserved},\\ \text{else}\\ \text{then }\omega_{k}⟶\bar{\omega_{k}}=\frac{\omega_{k}}{\sum_{i=1}^{n}\omega_{i}},\;k=1,2,\ldots ,n\text{ as the new weight},\\ \omega_{k}=\overset{⟶}{\omega_{k}}rg\overset{⟵}{\omega_{k}}. \end{array}$$

After the above steps, the subjective weight and objective weight of classifier *k* are used to obtain the combined weight by level difference maximization. Finally, it is judged whether the value range of the combined weight is satisfied. If the combined weight meets the condition, it will be retained; otherwise, it will be regularized.

## 4. Ensemble Learning Model Based on the ER Rule

**Figure 3:**
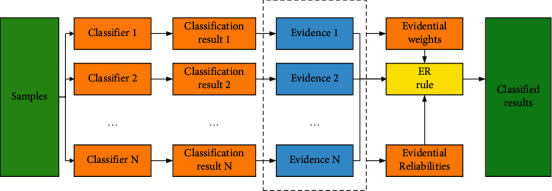
The process of ensemble learning based on the ER rule.

The method of setting the weight of evidence is defined in Section 3, the ensemble learning model based on ER rule is built in this section. When using the ER rule to combine classifiers in ensemble learning, each classifier obtains its classification results for the datasets, treats the classifiers as evidence in the ER rule, and calculates the weight and reliability of each piece of evidence. Decisions are made through the ER rule, and the classification results are calculated. This process is shown in Figure 3.

### 4.1. Setting the Reliability of Evidence

The reliability of evidence is an inherent characteristic of evidence, reflecting the ability of the evidence to provide correct assessments or solutions to hypotheses [30]. Based on the definition of evidence reliability, when using the ER rule for ensemble learning, each classifier in the process is regarded as independent evidence, and the classification accuracy of each classifier for the dataset is the ability to correctly evaluate the sample. Therefore, through mathematical statistics, the probability that a certain classifier is correctly classified for the dataset is calculated as its reliability, which is recorded as *r*_*k*_.

### 4.2. Setting the Weight of Evidence

**Figure 4:**
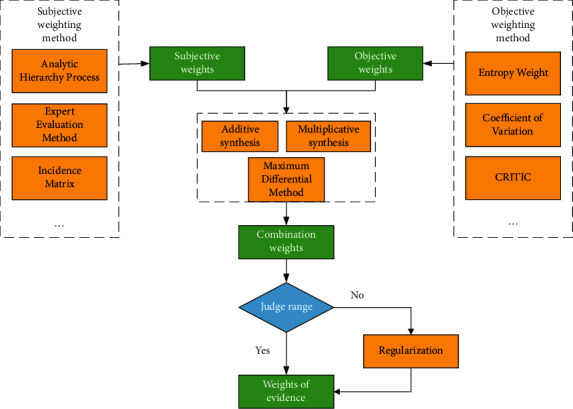
The process of weight of evidence assignment.

The weight of evidence is usually set by the subjective weighting method or objective weighting method, the specific steps of the weighting method are described in Section 3. This paper combines the two methods and proposes to use the combination weighting method to set weights for evidence. The process of assigning weight of evidence is shown in Figure 4. The AHP method in the subjective weighting method is combined with the EW method, COV method, and CRITIC method in the objective weighting method, and the results of the weighting are compared.

### 4.3. Evidential Reasoning Process

Assuming that each classifier in the ensemble process is an independent piece of evidence, there are *k* evidence in total. The category is considered as the evaluation level and the probability of the classifier's judgement on the sample category is considered as the belief level corresponding to the evaluation level. The reliability distribution of each piece of evidence can be expressed as

(20) $$\begin{array}{c} e_{k}=\left\{\left(\theta ,p_{\theta ,k}\right),\forall \theta \in \Theta ;\left(\Theta ,p_{\Theta ,k}\right)\right\}, \end{array}$$

where *θ* is the evaluation level, *p*_*θ*,*k*_ is the belief degree of the evaluation scheme evaluated as evaluation level *θ* under evidence *e*_*k*_, Θ is the discernment framework including all evaluation levels, *p*_Θ,*k*_ is the belief degree of indicator *k* relative to discernment framework Θ, namely, global ignorance, and *p*_Θ,*k*_ satisfies 0 ≤ *p*_*θ*,*k*_ ≤ 1, ∑_*θ*∈Θ_*p*_*θ*,*k*_ ≤ 1.

The reliability *r*_*k*_ and weight *ω*_*k*_ of the evidence have been determined by Section 4.1 and Section 3, respectively. The weighted belief distribution of the evidence *k* with reliability is

(21) $$\begin{array}{c} m_{k}=\left\{\left(\theta ,{\tilde{m}}_{\theta ,k}\right),\forall \theta \subseteq \Theta ;\left(P\left(\Theta \right),{\tilde{m}}_{P\left(\Theta \right),k}\right)\right\}, \end{array}$$

where *P*(Θ) is a power set and ${\tilde{m}}_{\theta ,k}$ is the mixed probability quality of indicator *k* under level *θ* and satisfies

(22) $$\begin{array}{c} \sum_{\theta \in \Theta }{\tilde{m}}_{\theta ,k}+{\tilde{m}}_{P\left(\Theta \right),k}=1,\\ {\tilde{m}}_{\theta ,k}=\left\{\begin{array}{c} 0,\theta =\emptyset ,\\ c_{rw,k}m_{\theta ,k},\theta \subseteq \Theta ,\theta \neq \emptyset ,\\ c_{rw,k}\left(1-r_{k}\right),\theta =P\left(\Theta \right), \end{array}\right.\\ {\tilde{m}}_{\theta ,k}=\left\{\begin{array}{c} 0,\theta =\emptyset ,\\ c_{rw,k}m_{\theta ,k},\theta \subseteq \Theta ,\theta \neq \emptyset ,\\ c_{rw,k}\left(1-r_{k},\right)\theta =P\left(\Theta \right), \end{array}\right. \end{array}$$

where *c*_*rw*,*k*_=1/(1+*ω*_*k*_−*r*_*k*_) is the regularization coefficient. *m*_*θ*,*k*_ is the basic probability quality of indicator *k* under level *θ*, and ∅ is an empty set.

Each classifier serves as an independent piece of evidence, and there are *K* pieces of evidence, so the combined belief *P*_*θ*,*e*(*b*)_ of *θ* is determined by the following equations for *b* pieces of evidence:

(23) $$\begin{array}{c} P_{\theta ,e\left(b\right)}=\left\{\begin{array}{c} 0,\theta =\emptyset ,\\ \frac{{\hat{m}}_{\theta ,e\left(b\right)}}{\sum_{A\subseteq \Theta }{\hat{m}}_{A,e\left(b\right)}},\theta \subseteq \Theta ,\theta \neq \emptyset , \end{array}\right.\\ m_{\theta ,e\left(b\right)}=\left\{\begin{array}{c} 0,\theta =\emptyset ,\\ \frac{{\hat{m}}_{\theta ,e\left(b\right)}}{\sum_{A\subseteq \Theta }{\hat{m}}_{A,e\left(b\right)}+{\hat{m}}_{P\left(\Theta \right),e\left(b\right)}},\theta \subseteq \Theta ,\theta \neq \emptyset , \end{array}\right.\\ {\hat{m}}_{P\left(\Theta \right),e\left(b\right)}=\left(1-r_{b}\right)m_{P\left(\Theta \right),e\left(b-1\right)},\\ {\hat{m}}_{\theta ,e\left(b\right)}=\left[\left(1-r_{b}\right)m_{\theta ,e\left(b-1\right)}+m_{P\left(\Theta \right),e\left(b-1\right)}m_{\theta ,b}\right]+\sum_{A\cap B=\theta }m_{A,e\left(b-1\right)}m_{B,b},\forall \theta \subseteq \Theta , \end{array}$$

where ${\hat{m}}_{\theta ,e\left(b\right)}$ is the degree of support for hypothesis *θ* of the combined result of the fusion of *b* pieces of independent evidence, and *b*=1,…, *K*, *m*_*θ*,*e*(1)_=*m*_*θ*,1_, and *m*_*P*(Θ),*e*(1)_=*m*_*P*(Θ),1_.

Supposing the utility of the evaluation level *θ* is *u*(*θ*), the expected utility *u* of the final model is

(24) $$\begin{array}{c} u=\sum_{\theta \in \Theta }u\left(\theta \right)P_{\theta ,e\left(b\right)}. \end{array}$$

Comparing the expected utility *u* with the utility *u*(*θ*) of the evaluation level, if *u*=*u*(*θ*), the result of the integration using the ER rule is the category *θ*.

## 5. Case Study

There are five types of datasets used in this study: part of a large fish dataset provided by the Kaggle platform, part of a flower recognition dataset, part of a fruit 360 dataset, Stanford's dog dataset, and part of the dataset from the public weather data package provided by the Baidu AI Studio platform. The specific parameter information of these five datasets is shown in Table 1.

The study steps are as follows:

1. First, we use five commonly used deep learning algorithms as the classifier, i.e., DenseNet121, MobileNetV2, InceptionV3, EfficintNet, and ResNet152V2, and use 60% of the samples in the dataset as the training set and all the samples as the test set for classification prediction. Three deep learning algorithms are randomly selected for integration from the five deep learning algorithms, with the number of samples as rows, and the combination of categories and deep learning algorithms as columns, forming a matrix of rowNum×12, where rowNum is the total number of samples in different datasets.
2. Second, we calculate the accuracy of the classifier for sample prediction as the reliability of the classifier. Taking the probability matrix as the original evaluation matrix, the subjective weights and the objective weights are calculated by the AHP, EW, COV, and CRITIC methods. These weights are combined by the addition, the multiplication and the level difference maximization to obtain the combined weight of the classifier.
3. Third, we take the classifier as the evidence, the category as the evaluation level of the evidence, and the probability predicted by different classifiers as the belief distribution. The ER rule is used to integrate the classifiers, the calculated expected utility value is compared with the evaluation level, and the judgement of the image category by the ER rule is calculated.
4. Fourth, the Spearman correlation analysis is used for statistical analysis of the results to determine whether there is a significant difference in performance between the combination weighting method and other weighting methods.

In this paper, the abbreviations D, M, I, E, and R refer to five classifiers DenseNet121, MobileNetV2, InceptionV3, EfficientNet, and ResNet152V2, respectively. Five classifier combinations, DIE, DIR, IER, MIE, and MIR, are used as study objects.

### 5.1. Study of Datasets

#### 5.1.1. Study Based on Large-Scale Fish Dataset

**Figure 5:**
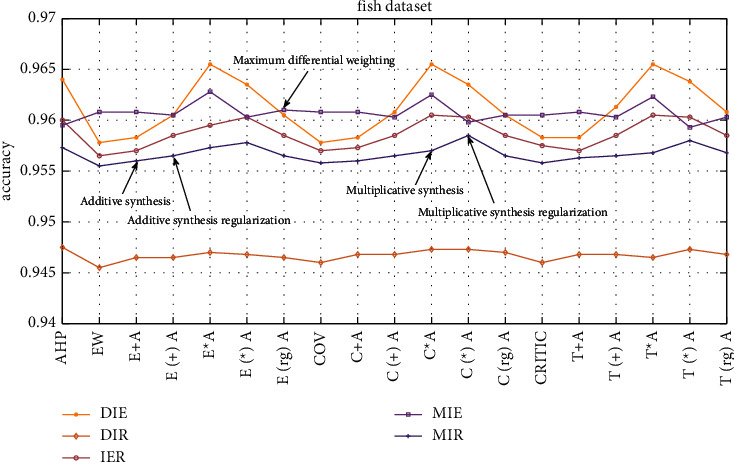
Comparison of ensemble accuracy of the big fish dataset.

The dataset is used in this study is part of the large fish dataset on the Kaggle platform. For each picture in the dataset, five classifiers are combined with the ER rule for ensemble learning, subjective and objective weighting methods are used to calculate weights and combined, weight regularization is considered, and the final accuracy is compared. The comparison results are shown in Table 2 and Figure 5. In Table 2, “+” means using the additive synthesis method for subjective and objective weights, “(+)” means performing regularization processing on the weights after using the additive synthesis method to combine weights, “*∗*” represents the multiplicative synthesis method, “(*∗*)” represents the regularization of the weights after combining the weights using the multiplicative synthesis method, and “(rg)” represents the weight by the level difference maximization method.

As shown in Table 2, the different methods used to determine weights have their own advantages and disadvantages in an ensemble. For example, when the three classifiers of DIR are combined for ensembles, the weight determined by the AHP method is the best, and the accuracy reaches 94.75%. Among the three combination methods of DIE, IER, and MIE, the weights determined by the multiplicative synthesis method are the best, and the highest accuracies of the three combination methods are 96.55%, 96.05%, and 96.28%, respectively. These five combinations are generally better than using subjective weighting and objective weighting alone to determine the weights by the level difference maximization method. In most cases, the combination weighting effect is significantly better. The bold values represent the maximum accuracy for each column of data.

As shown in Figure 5, in the MIR combination, the weights determined by the level difference maximization are generally better than the effect of using subjective weighting and objective weighting alone. After the weights determined by the additive synthesis method and the multiplicative synthesis method are regularized, all effects are better than those without regularization, and the accuracy is approximately 0.1% higher. For the other four combinations, the effect of regularization on the weights determined by the additive synthesis method is better than the effect without regularization, which is approximately 0.1%–0.3% higher. In addition, the level difference maximization method determines the weights close to the accuracy of the regularized additive synthesis method to determine the weights.

#### 5.1.2. Study Based on Weather Dataset

**Figure 6:**
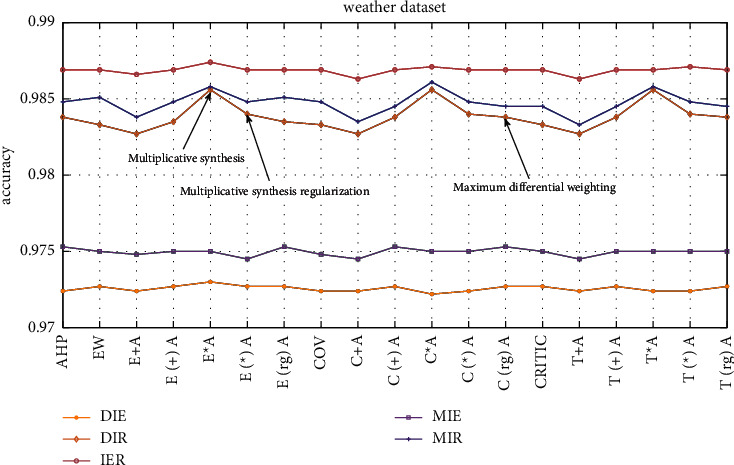
Comparison of ensemble accuracy of the weather dataset.

The dataset used in this study is part of the weather dataset on the Kaggle platform. For the images in the dataset, five classifiers and the ER rule are combined for ensemble learning, subjective and objective weighting methods and combination weighting methods are used to calculate weights, and the ER rule ensemble accuracies of different weight determination methods are compared. The comparison result is shown in Figure 6.

As shown in Figure 6, the multiplicative synthesis method of the four combinations of DIE, DIR, IER, and MIR has the best effect in determining the weights, and the accuracy is 97.30%–98.74%. The effect after regularization is better than the effect without regularization, and the accuracy is approximately 0.03%–0.11% higher. Sometimes the multiplicative synthesis method determines that the effect of the unregularized weight is better than the effect of the regularization, but in general, the effect of regularization is still better than the effect of nonregularization.

#### 5.1.3. Study Based on the Flower Dataset

**Figure 7:**
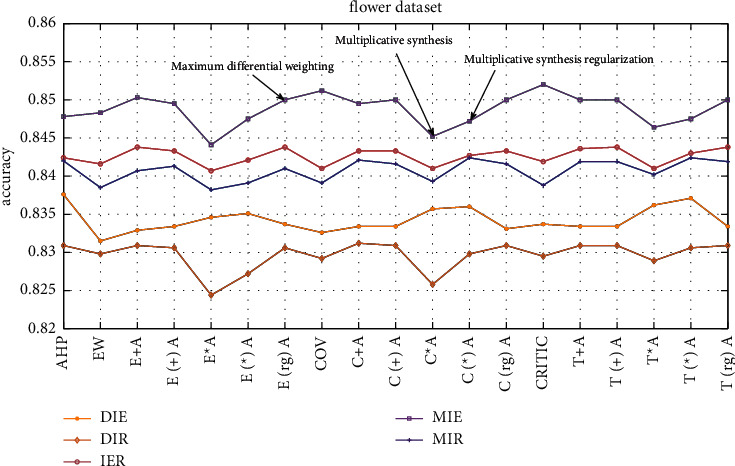
Comparison of ensemble accuracy of the flower dataset.

The dataset used in this study is part of the flower dataset on the Kaggle platform, and the content is pictures of various types of flowers. Five classifiers and the ER rule are combined for ensemble learning, and the ER rule ensemble accuracy of different weight determination methods is compared. The comparison result is shown in Figure 7.

As shown in Figure 7, under these five combinations, the effect of determining the weight of the level difference maximization method is generally better than other methods. The DIE combination has the best weight determination effect under the AHP method, with an accuracy of 83.76%. In addition, the regularized weight effect under this combination is better than the unregularized effect, the accuracy is increased by approximately 0.1%. The MIE combination has the best effect under the CRITIC method, with an accuracy rate of 85.20%. The effect after weight regularization is better than the effect without regularization, and the accuracy rate is approximately 0.03%–0.3% higher. Under the combination of MIR, the accuracy of additive synthesis and multiplicative synthesis to determine the regularized weights is approximately 0.05%–0.31% higher than that of nonregularization.

#### 5.1.4. Study Based on the Fruit Dataset

**Figure 8:**
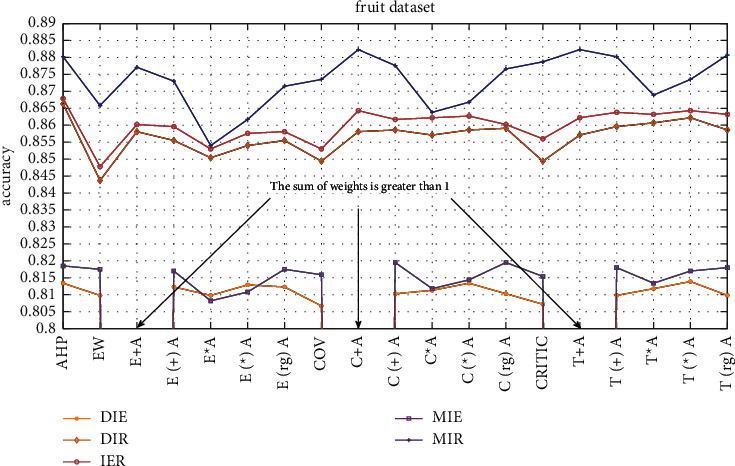
Comparison of ensemble accuracy of the fruit 360 dataset.

The dataset used in this study is part of the fruit 360 dataset on the Kaggle platform, and the content is pictures of different types of apples. For each picture, five classifiers and the ER rule are combined for ensemble learning, and the ER rule ensemble accuracies of different weight determination methods are compared. The comparison result is shown in Figure 8.

As shown in Figure 8, for the DIR combination, the effect of the level difference maximization method to determine the weight is generally better than other methods, and the additive synthesis method to determine the weight of the regularization effect is generally better than the effect of the nonregularization, and the accuracy is improved by approximately 0.06%–0.15%, the accuracy of the regularized weights determined by the multiplicative synthesis method is approximately 0.15%–0.36% higher than the accuracy of the unregularized. In the IER combination, the effect of weight regularization is generally better than that of nonregularization. But for the combination of DIE and MIE, there are three break points in Figure 7, because the weights determined by the additive synthesis method for the subjective and objective weights are greater than 1, which exceeds the range of weight of evidence in the ER rule, and the accuracy of the ensemble cannot be calculated. Compared with other points, the effect of regularization on the weight is generally better than the effect of nonregularization.

#### 5.1.5. Study Based on the Dog Dataset

The dataset used in this study is part of Stanford's dog dataset on the Kaggle platform, and the content is pictures of different kinds of dogs. For each picture, five classifiers and the ER rule are combined for ensemble learning, and the ER rule ensemble accuracies of different weight determination methods are compared. The comparison results are shown in Table 3.

As shown in Table 3, for the DIE combination, the level difference maximization method determines that the weighting effect is the best. Under the IER combination, the EW method, COV method, and EW (rg) AHP method determine the best weighting effect. In the MIE combination, the AHP method, CRITIC(+)AHP method, and CRITIC(+)AHP method have the best effect in determining the weight. In the MIR combination, the COV method has the best effect in determining the weight. However, for the four combinations of DIE, IER, MIE, and MIR, the range of weights determined when the additive synthesis method is used exceeds 1, which does not meet the range of weight of evidence in the ER rule, and the accuracy of the integration cannot be calculated, so replace it with “#“. In the DIR combination, the AHP method is used to determine the subjective weight. Since the final test coefficient *CR* is greater than 0.1, which does not meet the consistency test, the subjective weight cannot be calculated, and the combination weight cannot be calculated subsequently, so it is replaced with “&“. It can be concluded that the weights determined by the combination weighting method are not necessarily better than the weights determined by the subjective weighting method or the objective weighting method alone in all cases. The appropriate weighting method should be considered according to the actual situation. The bold values represent the maximum accuracy for each column of data.

According to the above cases, we can obtain the following conclusions:

1. First, in most cases, the combined weight determined by the level difference maximization has a better effect than the individual subjective and objective weights. The additive combination method and the multiplicative combination method also have a better effect. Compared with the individual subjective and objective weighting method, the combination weighting method has a significant improvement in the effect of the ensemble learning based on the ER rule. The accuracy of ensemble learning is improved by approximately 0.1%–0.4% through the use of combination weighting method in general. Therefore, using the combination weighting method to set the evidence weight of the ER rule has a better effect. Although the combination weighting method is effective, but additive combination method and the multiplicative combination method lack strict theoretical reasoning, so it is recommended to use the more theoretical method of the level difference maximization to combine the weights. The level difference maximization method is used as a combination weighting method for statistical analysis and comparative studies with other weighting methods in 5.2 and 5.3 Subsections.
2. Second, the effect of regularizing the weights on ensemble learning is generally better than that of nonregularization. The analysis of the level difference maximization method and the supporting evidence of the additive synthesis method and the multiplicative synthesis method show that the improvement of the ensemble learning accuracy of the regularization weight is approximately 0.01%–0.3% higher than that of the nonregularization weight. The regularization of weights can also prevent the combined weight from exceeding the range of the weight of evidence in the ER rule. In summary, the weights of evidence need to be regularized.
3. Third, the reasoning process of the ER rule is clear and the weight of evidence determined by the combination of weights is more reasonable, so the ensemble learning model based on the ER rule considering combination weighting is constructed on these basis is reasonable and universal. According to the experiments, this model can effectively improve the effect of ensemble learning.

### 5.2. Statistical Analysis

To show the differences in performance between the combination weighting method and the other weighting methods and verify the universality and effectiveness of the combination weighting method, we performed a statistical analysis between the different weighting methods.

The EW method, COV method, CRITIC method and combination weighting method including EW(rg)AHP, COV(rg)AHP, CRITIC(rg)AHP are tested for significance, and are denoted as “EW/EW(rg)AHP”, “COV/COV(rg)AHP”, “CRITIC/CRITIC(rg)AHP”. The AHP method and the best combination weighting method are tested for significance, and are denoted as “max(rg)”.

**Figure 9:**
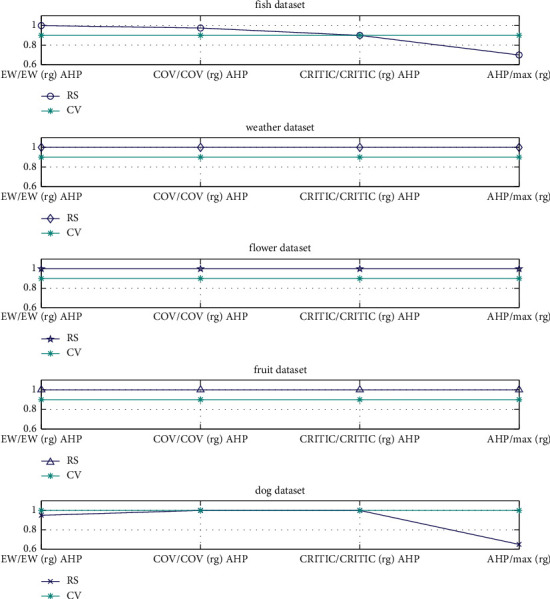
Comparison of significance levels of five datasets.

First, on the basis of the significance level parameter *α*=0 · 05, statistical analysis is performed for each method combination, and Spearman rank correlation coefficient is calculated. Second, comparing the Spearman rank correlation coefficient (*RS*) [31] with the Spearman rank correlation critical value (*CV*), if *RS* > *CV* proves that the differences in performance between the combination weighting method and the other methods is statistically significant. In the DIR combination in the dog dataset, the test coefficient *CR* of the AHP method is greater than 0.1 and the weight cannot be determined, and the combination is not analysed. Therefore, the number of combinations of this dataset is *n*=4, then *CV*=1; the number of combinations of other datasets is *n*=5, then *CV*=0 · 9. The comparison of significance levels is shown in Figure 9.

As shown in Figure 9, all the *RS* values are higher than *CV* in the weather dataset, flower dataset and fruit dataset; only the *RS* value of max(rg) is lower than *CV* in the fish dataset; and the *RS* values of EW/EW(rg)AHP and max(rg) are lower than *CV* in the dog dataset. It indicates differences in performance between the combination weighting method and the other methods is statistically significant.

To better judge that there are significant differences in performance between different methods, we also calculated *p* -values. The *p* -values are compared with the significance level, if *p* < 0.05 proves the differences in performance are statistically significant. Comparison of *p* values are shown in Table 4.

As shown in Table 4, most *p* values are less than 0.05, only the *p* values are greater than 0.05 in the dog dataset. Therefore, the differences in performance between the combination weighting method and other weighting methods is statistically significant.

### 5.3. Comparative Studies

To demonstrate the effectiveness of the combination weighting method in this paper, comparative studies are conducted in this subsection.

The combination weighting method not only considers the subjective intention of attribute decision-makers, but also contains the mathematical theoretical basis for objective weighting, so more reasonable weights of evidence can be determined. To further illustrate the universality and effectiveness of the above method, the order relation analysis method (G1) and the entropy weight method based on the rank sum ratio (RSR-EW) are selected as comparison methods [6, 12]. The level difference maximization methods are selected from the combination weighting method, including EW(rg)AHP, COV(rg)AHP, and CRITIC(rg)AHP. The accuracy of the ensemble learning model using the level difference maximization and the accuracy of the ensemble learning model using the comparative method are compared. The results of the comparison studies are shown in Table 5.

As shown in Table 5, the accuracy of the ensemble learning model using the level difference maximization method is higher than that of the ensemble learning model using the comparison method in most cases. Only the comparison method G1 is better in the fruit dataset. Therefore, it is reasonable and effective to use the combination weighting method to determine the weight of evidence, which can set a reasonable weight of evidence for the ensemble learning model based on the ER rule, thereby significantly improving the accuracy of the model.

## 6. Conclusion

In the field of ensemble learning based on the ER rule, problems include, a small number of classifiers in ensemble learning, difficulty in the quality judgement classifiers based on expert experience, objective weighting method that is too dependent on samples, and difficulty in setting a reasonable weight of evidence to improve the accuracy of ensemble learning. The combination weighting method is proposed to solve these problems. This method considers the subjective judgement of expert experience and the objective information of sample data, which overcomes the subjectivity or objectivity of the weight of evidence to a certain extent, making the weight of evidence more reasonable and significantly improve the recognition accuracy of the ensemble learning model. On this basis, the weight of evidence is regularized, which further improves the effect of ensemble learning model based on the ER rule.

There are three innovations in this paper. Firstly, in view of the problem that it is difficult to set a reasonable weight of evidence in traditional weighting methods, the combination weighting method is proposed to set more reasonable weights of evidence for the ER rule. Secondly, considering the lack of labels of the ER rule in the information fusion process, the weight of evidence is regularized. Lastly, based on setting the weight of evidence for the ER rule by using combination weighting method, the ensemble learning model based on the ER rule is constructed to improve the effect of integration.

Based on the above research, the main work in the future will include the following aspects: (1) In terms of the execution time of methods, the subjective weighting method is approximately 0.0003s-0.002s, the objective weighting method is approximately 0.03s-0.08s, and the combination weighting method is approximately 0.18s-0.32s, the execution time of the combination weighting method is longer. Therefore, how to reduce the execution time under the premise of ensuring the effect is the focus of future work. (2) This paper only studies the combination of several classic subjective weighting methods and objective weighting methods, and there are more combinations of combination weighting methods that can be used, such as game theory.

## Figures and Tables

**Table 1:** Dataset parameters.

Dataset name	Total number of pictures	Number of categories
Large-scale fish dataset	4000	4
Weather dataset	3882	4
Flowers recognition dataset	3557	4
Fruits 360 dataset	1945	4
Stanford's dog dataset	639	4

**Table 2:** Ensemble accuracy of the big fish dataset.

	DIE	DIR	IER	MIE	MIR
EW	0.9578	0.9455	0.9565	0.9608	0.9555
COV	0.9578	0.9460	0.9570	0.9608	0.9558
CRITIC	0.9583	0.9460	0.9575	0.9605	0.9558
AHP	0.9640	**0.9475**	0.9600	0.9595	0.9573
EW + AHP	0.9583	0.9465	0.9570	0.9608	0.9560
COV + AHP	0.9583	0.9468	0.9573	0.9608	0.9560
CRITIC + AHP	0.9583	0.9468	0.9570	0.9608	0.9563
EW*∗*AHP	**0.9655**	0.9470	0.9595	**0.9628**	0.9573
COV*∗*AHP	**0.9655**	0.9473	**0.9605**	0.9625	0.9570
CRITIC*∗*AHP	**0.9655**	0.9465	**0.9605**	0.9623	0.9568
EW(+)AHP	0.9605	0.9465	0.9585	0.9605	0.9565
COV(+)AHP	0.9608	0.9468	0.9585	0.9603	0.9565
CRITIC(+)AHP	0.9613	0.9468	0.9585	0.9603	0.9565
EW(*∗*)AHP	0.9635	0.9468	0.9603	0.9603	0.9578
COV(*∗*)AHP	0.9635	0.9473	0.9603	0.9598	**0.9585**
CRITIC(*∗*)AHP	0.9638	0.9473	0.9603	0.9593	0.9580
EW(rg)AHP	0.9605	0.9465	0.9585	0.9610	0.9565
COV(rg)AHP	0.9605	0.9470	0.9585	0.9605	0.9565
CRITIC(rg)AHP	0.9608	0.9468	0.9585	0.9603	0.9568

**Table 3:** Ensemble accuracy of Stanford's dog dataset.

	DIE	DIR	IER	MIE	MIR
EW	0.8701	0.8858	**0.8905**	0.8779	0.8905
COV	0.8748	0.8826	**0.8905**	0.8842	**0.8951**
CRITIC	**0.8811**	0.8842	0.8858	**0.8920**	0.8936
AHP	**0.8811**	&	0.8826	0.8811	0.8858
EW + AHP	#	&	#	#	#
COV + AHP	#	&	#	#	#
CRITIC + AHP	#	&	#	#	#
EW*∗*AHP	0.8748	&	0.8842	0.8748	0.8826
COV*∗*AHP	0.8764	&	0.8826	0.8764	0.8811
CRITIC*∗*AHP	0.8795	&	0.8858	0.8764	0.8811
EW(+)AHP	0.8764	&	0.8889	0.8826	0.8920
COV(+)AHP	0.8764	&	0.8889	0.8873	0.8920
CRITIC(+)AHP	0.8795	&	0.8858	**0.8920**	0.8936
EW(*∗*)AHP	0.8748	&	0.8811	0.8748	0.8811
COV(*∗*)AHP	0.8795	&	0.8826	0.8779	0.8826
CRITIC(*∗*)AHP	**0.8811**	&	0.8811	0.8826	0.8826
EW(rg)AHP	0.8764	&	**0.8905**	0.8858	0.8920
COV(rg)AHP	0.8764	&	0.8889	0.8873	0.8920
CRITIC(rg)AHP	0.8795	&	0.8873	**0.8920**	0.8936

**Table 4:** Comparison of *p* values.

Dataset	EW/EW(rg)AHP	COV/COV(rg)AHP	CRITIC/CRITIC(rg)AHP	AHP/max(rg)
Fish	0.0167	0.0333	0.0833	0.2333
Weather	0.0167	0.0167	0.0167	0.0167
Flower	0.0167	0.0167	0.0167	0.0167
Fruit	0.0167	0.0167	0.0167	0.0167
Dog	0.1667	0.0833	0.0833	0.5

**Table 5:** Comparative study of five datasets.

Dataset	Classifier	Combination weighting method			Comparative method	
		EW(rg)AHP	COV(rg)AHP	CRITIC(rg)AHP	RSR-EW	G1
Fish	DIE	0.9605	0.9605	0.9608	0.9608	0.9625
	DIR	0.9465	0.9470	0.9468	0.9468	0.9470
	IER	0.9585	0.9585	0.9585	0.9578	0.9590
	MIE	0.9610	0.9605	0.9603	0.9608	0.9603
	MIR	0.9565	0.9565	0.9568	0.9533	0.9563

Weather	DIE	0.9727	0.9727	0.9727	0.9719	0.9724
	DIR	0.9835	0.9838	0.9838	0.9830	0.9838
	IER	0.9869	0.9869	0.9869	0.9866	0.9869
	MIE	0.9753	0.9753	0.9750	0.9742	0.9750
	MIR	0.9851	0.9845	0.9845	0.9835	0.9845

Flower	DIE	0.8337	0.8331	0.8334	0.8331	0.8346
	DIR	0.8306	0.8309	0.8309	0.8275	0.8301
	IER	0.8438	0.8433	0.8438	0.8410	0.8433
	MIE	0.8500	0.8500	0.8500	0.8512	0.8401
	MIR	0.8410	0.8416	0.8419	0.8376	0.8410

Fruit	DIE	0.8123	0.8103	0.8098	0.8041	0.8093
	DIR	0.8555	0.8591	0.8586	0.8504	0.8612
	IER	0.8581	0.8602	0.8603	0.8581	0.8658
	MIE	0.8175	0.8195	0.8180	0.8149	0.8201
	MIR	0.8715	0.8766	0.8807	0.8823	0.8828

Dog	DIE	0.8764	0.8764	0.8795	0.8811	0.8842
	DIR	&	&	&	&	&
	IER	0.8905	0.8889	0.8873	0.8936	0.8842
	MIE	0.8858	0.8873	0.8920	0.8842	0.8889
	MIR	0.8920	0.8920	0.8936	0.8905	0.8936

## Data Availability

The study data in this paper come from the image classification datasets of the Kaggle platform and Baidu AI Studio platform. For the source URL of the datasets, please visit: (1) Large-scale Fish dataset: https://www.kaggle.com/crowww/a-large-scale-fish-dataset. (2) Weather dataset: https://aistudio.baidu.com/aistudio/datasetdetail/13165. (3) Flowers recognition dataset: https://www.kaggle.com/alxmamaev/flowers-recognition. (4) Fruits 360 dataset: https://www.kaggle.com/moltean/fruits. (5) Stanford's dogs dataset: https://www.kaggle.com/jessicali9530/stanford-dogs-dataset
